# *VDR* Gene Polymorphisms and Diffuse Large B-Cell Lymphoma: Correspondence

**DOI:** 10.31557/APJCP.2026.27.2.401

**Published:** 2026-02-05

**Authors:** Amnuay Kleebayoon, Viroj Wiwanitkit

**Affiliations:** 1 *Private Academic Consultant, Samraong, Cambodia. *; 2 *Department of Research Analytics, Saveetha Dental College and Hospitals, Saveetha Institute of Medical and Technical Sciences Saveetha University, India. *

**Dear Editor**

The publication on “Association of Selective *VDR* Gene Polymorphisms with Diffuse Large B-Cell Lymphoma (DLBCL) in a South Indian Population [1]” is interesting. This study is an intriguing attempt to link genetic anomalies (specifically, *VDR* gene polymorphisms) to the risk of diffuse large B-cell lymphoma (DLBCL) in a South Indian community. However, there are various limitations that may impair the reliability and interpretation of the findings. For example, the study’s cross-sectional design does not allow for clear cause and effect, and the sample size of only 50 patients and 100 controls may be insufficient to draw population-level conclusions, particularly when performing complex genetic analyses that require larger sample sizes to account for genetic variability.

## Questions to stimulate discussion include

Are the protective associations of mutant alleles, such as TT-BsmI and AG-TaqI with DLBCL risk the result of clear biological mechanisms, or are they simply statistical associations caused by sampling bias or linkage disequilibrium with other genes? Another question is whether the effects of these *VDR* polymorphisms correlate with vitamin D levels in the body, which is significant given that vitamin D levels were not examined in this sample.

Although the data show that some SNPs in the *VDR* gene are related with a lower incidence of DLBCL, further interpretations suggest that this association may result from a population-specific immunological variation mechanism rather than a direct effect of vitamin D. For example, the C-A-G-T haplotype identified to be associated with disease could be linked to the expression of genes that govern B- or T-cells at a deeper level than just vitamin D response. Furthermore, comparisons with other populations demonstrate the significance of ethnic context in genetic research.

This study concentrated on SNPs in the *VDR* gene; however, in relation to DLBCL a condition linked to immune dysfunction and cell division regulation it is conceivable that polymorphisms in other genes related to immunity, such as *IL-10, TNF-α, IL-6,* or those governing B-cell proliferation (e.g., *BCL2, MYC, P53*), could also be significant [2]. Failure to account for these polymorphisms may result in overlooking deeper connections between genetics and disease pathology. Moreover, prior research suggests that the* CYP27B1* and *CYP24A1* genes, which are involved in the synthesis and metabolism of vitamin D, may contribute to immune regulation through active vitamin D (1,25(OH)₂D) [3]. To achieve a more thorough understanding of the vitamin D and immune system, future studies should broaden their scope to include these genes.

To broaden the perspective, multivariate regression studies incorporating other characteristics such as age, gender, nutritional status, or body mass index should be conducted to see whether the effect of *VDR* polymorphisms remains significant when these covariates are controlled. Furthermore, measuring blood vitamin D levels in conjunction with SNP analysis will assist answer the critical question of whether the presence of a mutant allele impacts vitamin D activity in clinical settings. Finally, future research should use prospective cohort designs to better understand the long-term relationship between *VDR* polymorphisms and DLBCL prognosis.
