# Supplementary material for: VDR Gene Polymorphisms and Diffuse Large B-Cell Lymphoma: Correspondence
Source: Asian Pac J Cancer Prev. 2026 Feb 5;27(2):401–2. doi: 10.31557/APJCP.2026.27.2.401 (PMC13499979; doi:10.31557/APJCP.2026.27.2.401)
Supplement: Related texts

# *Reply to the letter to the editor: VDR *Gene Polymorphisms and Diffuse Large B-Cell Lymphoma: correspondence

**Published:** 2026-02-05

**Authors:** Annuja Anandaradje, Sandhiya Selvarajan

**Affiliations:** 1 *Department of Clinical Pharmacology, JIPMER, Puducherry. *

**Dear Editor**

We thank you for the interest shown towards our article titled “Association of Selective *VDR* Gene Polymorphisms with Diffuse Large B-Cell Lymphoma (DLBCL) in a South Indian Population” and appreciate the suggestions provided for further discussion.

## 1. VDR gene association with DLBCL

Our findings suggest that polymorphisms in BsmI, TaqI, and FokI of the *VDR* gene may be associated with DLBCL in the south Indian population. We acknowledge that the cross-sectional design and the modest sample size of 50 patients and 100 controls warrant cautious interpretation of the association between SNPs and disease risk. We recognize the need for larger, multicentric studies and targeted sequencing of the *VDR* gene to validate these results. Nonetheless, our study results suggest an indication towards the possible role of *VDR* genes in disease association. These preliminary findings may help future studies aimed at establishing causality using a larger sample size and a cohort design.

## 2. Genes linked to vitamin D metabolic pathway

We appreciate the thoughtful suggestion to include other immune-regulatory genes, such as IL-6, IL-10, TNF-α, BCL2, MYC, and P53, in future genetic analyses. However, the present study, which was designed to explore the association of various polymorphisms in *VDR* gene, did not consider other immune regulated genes during conception, considering feasibility and financial constraints. That said, the suggestion is well taken and such an investigation could be conducted as an independent study in future. Further, as rightly pointed out about the genes linked to synthesis and metabolism of vitamin D to achieve thorough understanding of vitamin D, we have already completed a study in DLBCL patients with vitamin D pathway genes which is yet to be published.

## 3. Correlation of VDR polymorphisms with vitamin D level

As rightly pointed out, our findings suggest a statistically significant association between the SNPs (BsmI, FokI and TaqI) with DLBCL susceptibility. Similarly, Purdue et al. [1], reported association of FokI, BsmI and TaqI polymorphisms in *VDR* with few subtypes of NHL. Although such associations may occasionally be due to linkage disequilibrium with other functional variants or sampling variability, existing literature does support a functional role for *VDR* polymorphisms in DLBCL through modulation of immune responses, apoptosis, and cell proliferation, a process central to lymphomagenesis [2, 3]. However, further studies are required to explore whether genotype and haplotype associations are driven by population specific immunological variation mechanism rather than a direct effect of vitamin D.

## 4. Multivariate regression

We agree that adjusting for relevant covariates through multivariate regression would enhance the robustness of future analyses, allowing for a clearer understanding of whether *VDR* polymorphisms independently contribute to DLBCL risk. Nevertheless, this was not attempted in our study due to the small sample size.

## 5. Future research

We agree that future research employing a prospective cohort design and whole genome sequencing will provide a better understanding of the association between genetic polymorphisms and DLBCL susceptibility.

## References

[1] Purdue MP, Lan Q, Kricker A, Vajdic CM, Rothman N, Armstrong BK (2007). Vitamin d receptor gene polymorphisms and risk of non-hodgkin’s lymphoma. Haematologica..

[2] Denzer N, Vogt T, Reichrath J (2011). Vitamin d receptor (VDR) polymorphisms and skin cancer: A systematic review. Dermatoendocrinol..

[3] Campbell MJ, Trump DL (2017). Vitamin d receptor signaling and cancer. Endocrinol Metab Clin North Am..
